# A novel quantitative PCR mediated by high-fidelity DNA polymerase

**DOI:** 10.1038/s41598-017-10782-4

**Published:** 2017-09-04

**Authors:** Mengling Zhang, Kyle Liu, Yihong Hu, Yi Lin, Yang Li, Ping Zhong, Xia Jin, Xiaoli Zhu, Chiyu Zhang

**Affiliations:** 1 School of Life Sciences, Shanghai University, Shanghai, China; 2 Pathogen Diagnostic Center, CAS Key Laboratory of Molecular Virology & Immunology, Institut Pasteur of Shanghai, Chinese Academy of Science, Shanghai, China; 3 Shanghai Municipal Center for Disease Control & Prevention, Shanghai, China; 4 Viral Disease and Vaccine Translational Research Unit, CAS Key Laboratory of Molecular Virology & Immunology, Institut Pasteur of Shanghai, Chinese Academy of Sciences, Shanghai, China

## Abstract

The biggest challenge for accurate diagnosis of viral infectious disease is the high genetic variability of involved viruses, which affects amplification efficiency and results in low sensitivity and narrow spectrum. Here, we developed a new simple qPCR mediated by high-fidelity (HF) DNA polymerase. The new method utilizes an HFman probe and one primer. Fluorescent signal was generated from the 3′–5′ hydrolysis of HFman probe by HF DNA polymerase before elongation initiation. Mismatches between probe/primer and template have less influence on the amplification efficiency of the new method. The new qPCR exhibited higher sensitivity and better adaptability to sequence variable templates than the conventional TaqMan probe based-qPCR in quantification of HIV-1 viral load. Further comparison with COBAS TaqMan HIV-1 Test (v2.0) showed a good correlation coefficient (R^2^ = 0.79) between both methods in quantification of HIV-1 viral load among 21 clinical samples. The characteristics of tolerance to variable templates and one probe-one primer system imply that the probe/primer design for the new method will be easier and more flexible than the conventional method for highly heterogeneous viruses. Therefore, the HF DNA polymerase-mediated qPCR method is a simple, sensitive and promising approach for the development of diagnostics for viral infectious diseases.

## Introduction

Real-time quantitative PCR (qPCR) was developed in early 1990s for the detection and quantification of nucleic acids^1^, which is widely used in almost all fields of biomedical research, agriculture, food and environment sciences^2–5^. Although some new methods such as isothermal amplification methods, loop mediated isothermal amplification (LAMP)^6, 7^, rolling circle amplification (RCA)^8, 9^, recombinase polymerase amplification (RPA)^10^ and specific high-sensitivity enzymatic reporter unlocking (SHERLOCK)^11^ had been developed in recent years, qPCR is still considered to be the most robust technology in clinical diagnostic assays, especially for infectious diseases^12–14^. RT-qPCR is usually accomplished by using either double-stranded DNA-binding dyes (e.g. SYBR green I^15^ and SYTO9^16, 17^ or fluorescence-based probes^18^. The dsDNA-binding dyes are cheaper than reagents used in the probe-based methods; however, their non-specific binding to dsDNA may result in false positive results, and inaccurate quantification^19^, and thus limits their use in qPCR for qualification of gene expression and molecular diagnosis. There are three main types of fluorescence-based probes in qPCR, including hydrolysis probes (often called as TaqMan probes)^20^, hybridization probes^21, 22^, and molecular beacons^23^. Some other types of probes, such as scorpion primers^24^, light upon extension (LUX) primers^25^ and Amplifluor primers^26^ were also developed for qPCR. Among these new probe types, the TaqMan probes were the most widely used for the detection and quantification of nucleic acids, and often developed into commercial kits for clinical diagnosis. Molecular beacons were more often used in the detection of single nucleotide polymorphisms (SNP)^27, 28^.

The detection and quantification of pathogens requires high sensitivity, specificity and reproducibility^29^. For qPCR, high sensitivity and specificity depend on the well-designed primers and probe that all bind to the highly conserved genomic regions of target pathogens. If the primers or probe bind to non-conserved genomic (e.i. highly variable) regions, amplification efficiency will be greatly reduced, which is a major reason for low detection sensitivity and specificity for some viruses^29, 30^. Currently, emerging and reemerging infectious diseases are a serious threat to global public health. More than 70% of emerging and reemerging infectious diseases are caused by viruses, and vast majority of human viruses (especially RNA viruses) have higher mutation rates than bacteria, fungi and other pathogens (e.g. mycoplasma and chlamydia). Because of high genetic variability, some viruses (e.g. HIV-1, HCV) are divided into genotypes or subtypes^30–32^. Therefore, it is more difficult to find highly conserved regions shared by all genotypes or subtypes of the same virus. It is harder to design and select appropriate qPCR primers and probes for virus detection with high detection sensitivity and specificity than for bacteria, fungi and other pathogens^29^. Furthermore, the qPCR requires a pair of primers and a TaqMan probe, and the probe length usually requires more than 25 nt to ensure relatively high Tm value (68–70 °C)^2, 18^, which further increase the difficulty of primer and probe design. Although MGB (minor groove binder) modification had been demonstrated to increase the stability of duplexes formed by probe and target, and allows shorter probes^33^, it is still a big challenge for the development of qPCR methods for high-efficient detection and quantification of all genotypes/subtypes of highly heterogenetic viruses such as HIV-1 and HCV.

In this paper, we developed a new simple qPCR method that is mediated by high-fidelity DNA polymerase and utilizes a primer and a fluorescent primer (called as HFman probe). The new method allows the presence of mismatches between the 3-ends of the HFman probe/primer and the target sequence, it has better performance in quantification of gene expression and viral load than the conventional TaqMan probe-based qPCR. Tolerance to mismatches between primer/probe and target and the one primer-one HFman probe system simplified the method, improved the sensitivity and accuracy for the quantification of gene expression, and diagnosis of various pathogens (especially for viruses with high heterogeneity).

## Results

### The principle of the novel real-time RT-PCR mediated by high-fidelity DNA polymerase

**Figure 1:**
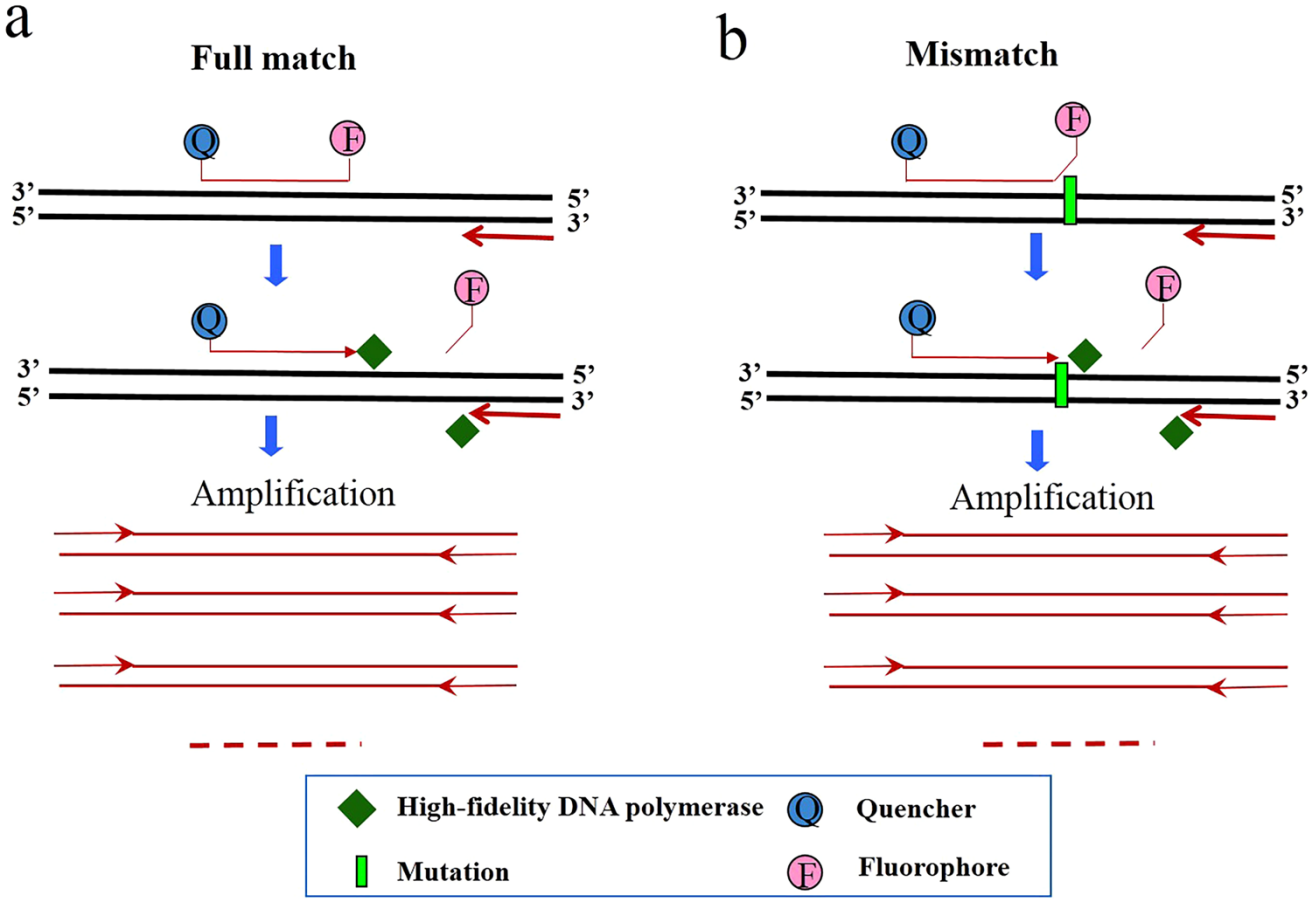
The principle of the novel real-time fluorescent PCR. (**a**) When the HFman probe fully matches with the template, the high-fidelity DNA polymerase can non-specifically recognize 3′ fluorophore-modified base and remove the 3′-base to initiate primer extension. (**b**) When the HFman probe forms a mismatch with the template at the 3′-end, the high-fidelity DNA polymerase recognize the 3′ mismatched base and remove the mismatched base to initiate extension. The removal of 3′ fluorophore-labelled base will emit the fluorescent signal.

High-fidelity DNA polymerase has a 3′–5′ exonuclease activity to remove the mismatched bases from the 3′-end of a newly synthesized DNA strand or primer. Various chemical modifications (e.g. phosphorylation, amination, and dideoxy nucleotide) at 3′-end base or the 3′-OH of the primer do not influence the removing (proof-reading) activity of the HF DNA polymerase^34^. A fluorescent probe (e.g. TaqMan probe) often contains a fluorophore and quencher group at 5′ and 3′ end of an oligonucleotide, respectively. The fluorophore- or quencher-modified base can be removed from the probe by HF DNA polymerase when the 3′-base forms a mismatch with the template (Fig. 1). In fact, some HF DNA polymerase can non-specifically remove the 3′ modified or unmodified base and start extension even though no mismatch occurs^35^. The removal of the fluorophore or quencher group from the probe results in the release of the fluorescent signal. Therefore, a probe can be used directly as a primer to initiate PCR amplification under the HF DNA polymerase and the emitted fluorescence during the amplification can be monitored during a real-time PCR amplification (Fig. 1). We call the new method as HF DNA polymerase mediated real-time PCR and defined the probe as HFman probe or fluorescent primer (Fig. 1). Compared with the conventional qPCR that requires a pair of primers and a probe, the new method needs only one HFman probe and one corresponding primer.

To prove the viability of the new method, we designed an HFman probe (N9-probe-1) and a primer for amplification of neuraminidase (NA) gene of H7N9 virus, and performed the new RT-qPCR assay. The N9-probe-1 has a fluorophore group at the 5′ end. As expected, an S-shape amplification curve was observed in the positive reaction but not in the negative control (data not shown), indicating the new method works.

**Figure 2:**
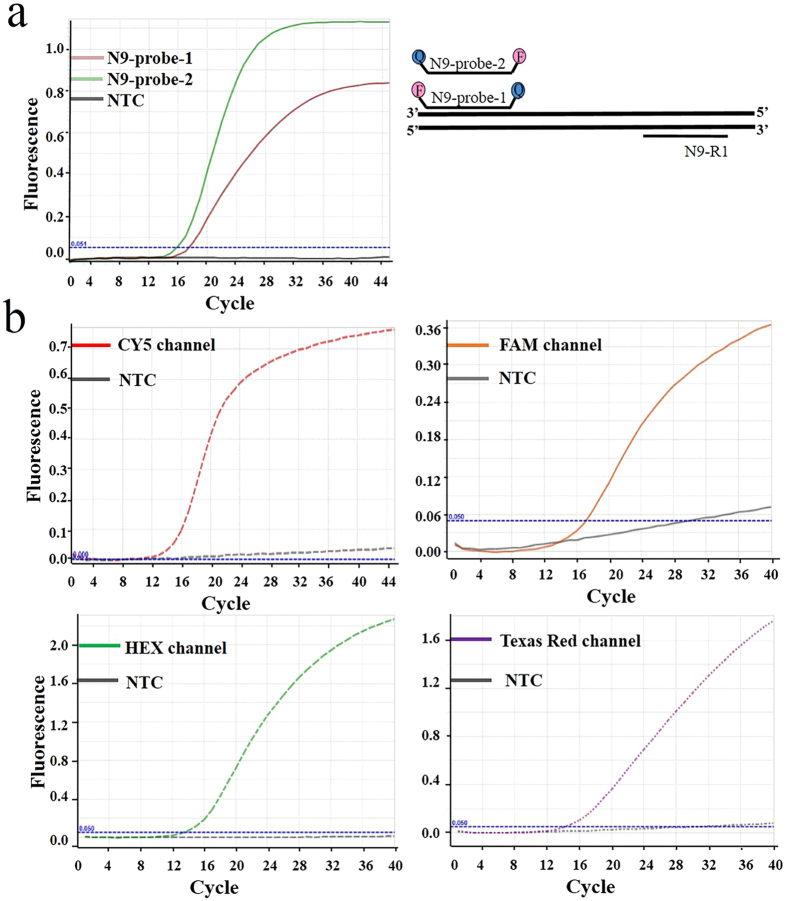
Performance of the novel method using probes labelled by different fluorescent dyes. (**a**) Comparison between the probes with 3′- and 5′ fluorophore groups. Both probes were labelled by FAM. (**b**) Performance of the novel method using probes labelled by four different fluorescent dyes, HEX, FAM, CY5 and Texas Red. Four fluorescent dyes can be detected in four different fluorescent channels. NTC, non-template control.

In the conventional qPCR, because the cleavage of TaqMan probe is from 5′ to 3′ by Taq DNA polymerase, the fluorophore group is usually labelled at the 5′ end of the probe to increase the emission of fluorescence. For HFman probe, fluorophore group is allowed to be labelled at either 5′ end or 3′end. However, because only bases at the 3'-end of HFman probe can be removed by HF DNA polymerase, a 3′ fluorophore labelling is thought to be more efficient than a 5′ fluorophore labelling. To prove this hypothesis, we designed N9-probe-2 that has a fluorophore group at the 3′ end, and performed a comparison between N9-probe-1 and N9-probe-2. The result showed that the reaction with N9-probe-2 had an earlier amplification curve with stronger fluorescence signal than that with N9-probe-1 (Fig. 2a), supporting a HFman probe with a 3′-fluorophore group is more efficient than that with 5′-fluorophore group.

RT-qPCR can be carried out in singleplex or multiplex formats. Multiplex RT-qPCR has more widespread application than a singleplex format in molecular diagnostics of infectious diseases (especially for co-infection and multiple infections). To test the possibility of using the new qPCR method in a multiplex format, we designed a *β-actin*-specific HFman probe that was labelled individually with four different fluorescent dyes at 5′ end. Four fluorescent dyes HEX, FAM, CY5, and Texas Red were labelled at 5′ end with four quenchers BHQ1, TAMRA, BHQ3, Eclipse at 3′end, respectively (Supplementary Table S1). The four dyes can emit fluorescence signals at different wavelengthes that are monitored in different fluorescent channels, and are widely used in the Multiplex RT-qPCR assay. Four good amplification curves were observed in different fluorescent channels (Fig. 2b), indicating that HF DNA polymerase recognize HFman probe labelled with different fluorophore and quencher groups. These results imply that the new qPCR can be developed into a multiplex format.

### Optimization of the reaction system

To assess the performance of various HF DNA polymerases and select the best one for the new qPCR method, we tested seven different commercially available HF DNA polymerases. Although various enzymes have different fidelities, all seven HF DNA polymerases worked in the new qPCR system (Supplementary Fig. S1). The Q5 enzyme was selected for subsequent experiments because of more rapid amplification (Supplementary Fig. S1).

To obtain the best performance of the new qPCR, we first optimized the concentrations of MgCl_2_ by adding 0 to 4 mM of MgCl_2_. The results showed that increasing amount of MgCl_2_ enhanced the total fluorescence signal intensity at the amplification plateau period, but did not improve the amplification efficiency (Supplementary Fig. S2a). We selected 3 mM MgCl_2_ for subsequent analysis. We further optimized the concentration and proportion of HFman probe to the primer. The concentrations of HFman probe and primer were from 0.1–0.4 µM and 0.2–0.4 µM, respectively, and the proportions of HFman probe to the primer were from 1:1 to 1:4 (Supplementary Fig. S2b). The result show that the reaction with 0.1 µM HFman probe and 0.4 µM primer had earlier amplification curves (Supplementary Fig. S2b). Therefore, 0.1 µM HFman probe and 0.4 µM primer were recommended for the new method.

### Flexibility of the new qPCR method to probe and/or primer mismatches

RT-qPCR has a very wide use especially in diagnostics of infectious diseases. About 70% infectious diseases are caused by viruses that have higher mutation rates than bacteria and other non-viral pathogens. Therefore, it is difficult to find absolutely conserved genomic regions of some viruses (e.g. HIV-1) for primer and probe design. The presence of mismatch between primer/probe and template reduces the amplification efficiency and sensitivity of RT-qPCR, although the use of degenerate primers can slightly improve the assay. The principle of the new qPCR method mediated by the HF DNA polymerase requires the presence of mismatch to initiate the 3′-5′ exonuclease activity of HF enzyme for removing the 3′ fluorophore- or quencher-modified base from the probe. Therefore, the new RT-qPCR is essentially resistant to mutations existent in templates.

**Figure 3:**
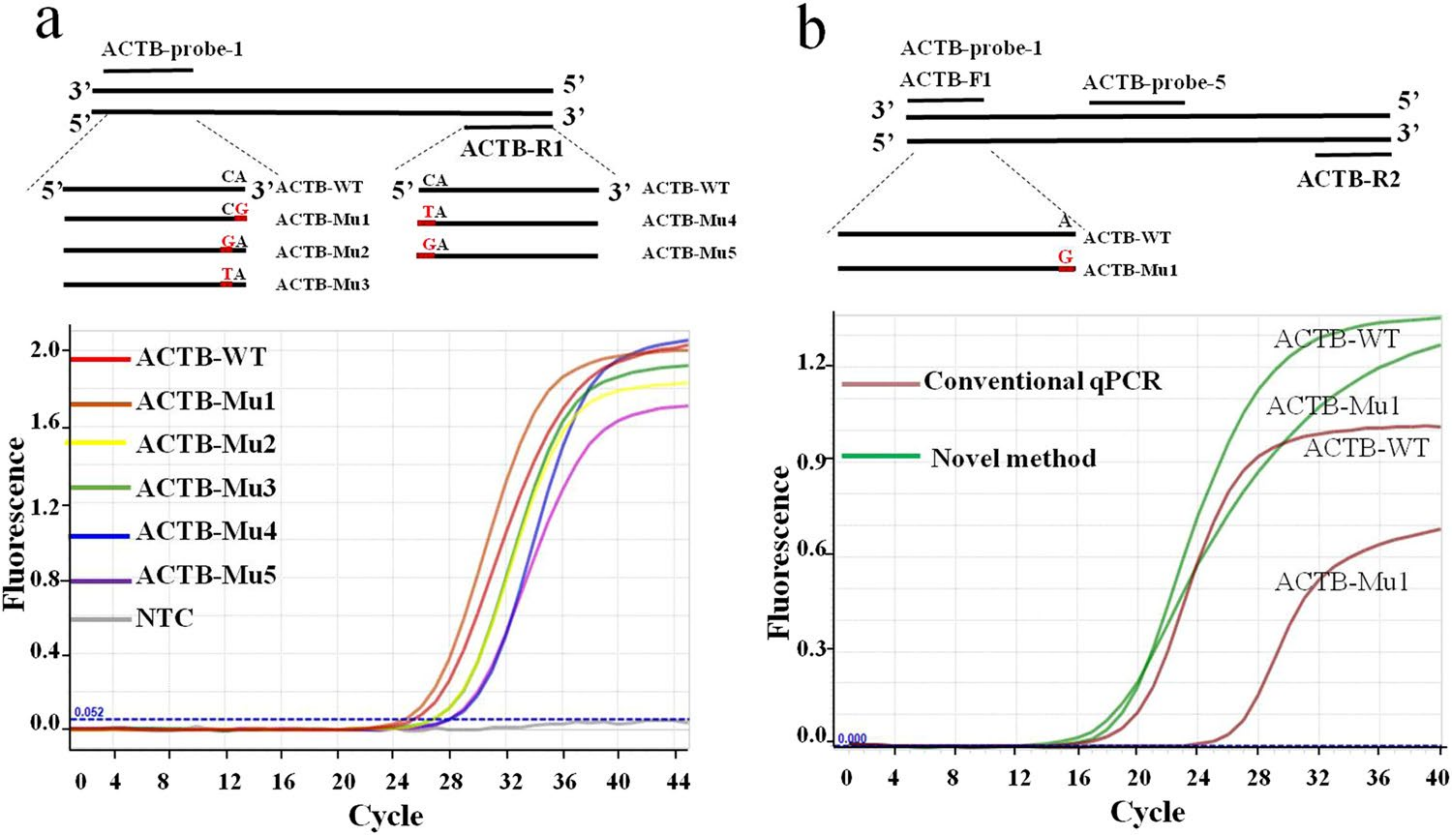
The influence of various mismatches between probe/primer and template on the amplification of the novel method. (**a**) The influence of various mismatches on the amplification of the novel method. The results of some other mismatches are shown in Supplementary Table S1. (**b**) Comparison between the novel method and the conventional qPCR in amplification of mismatched templates. The probe and primers fully match with the vile-type template. WT: wild type; Mu: mutant. The sequence information of probes and primers are shown in the Supplementary Table S1.

To address this hypothesis, we designed and constructed several mutant *β-actin* RNA templates that form mismatches with the 3′-end of the HFman probe or primer (Supplementary Table S1). All mutants and wild-type can be amplified with a slightly different efficiency (Fig. 3a). The template without mismatch and with one mismatch at the 3′-last base of the probe showed slightly higher amplification efficiency than those with a mismatch at the second last 3′-base of the probe. The templates forming mismatch with HFman probe have slightly higher efficiency than those forming mismatch with the reverse primer, possibly due to the mismatch between reverse primer and RNA templates which limits the speed of reverse transcription. To further test the effect of various mismatches between HFman probe and template on amplification efficiency, we synthesized four HFman probe with different bases at 3-end (Probe-A, -G, -C, and -T), and constructed four RNA templates that completely match to each of the probes (Supplementary Fig. S3). The probe has HEX and BHQ1 in the 5′ and 3′ end, respectively. The templates carrying C and G at the position corresponding to 3′-end of HFman probe have slightly lower amplification efficiency (higher Ct values) than templates carrying A and T (Supplementary Fig. S3) for four probes.

To further demonstrate the flexibility of the new RT-qPCR method to variants, we performed a comparison between the new method and the conventional (TaqMan probe-based) RT-qPCR method. To increase the comparability, the forward primer of the conventional method shares identical sequence with the HFman probe, and an additional TaqMan probe was designed for the conventional method. For the wild template, although the curve of the new method appeared slightly earlier and had stronger fluorescent signal than that of the conventional method, the amplification curves produced by both methods are close to each other (Ct: 23.56 vs. 27.58), supporting the idea that both methods are comparable. When the mutant template was used, the curve (Ct = 17.98) produced by the new method was obviously earlier and the fluorescent signal was substantially stronger than that by the conventional method (Ct = 26.35) (Fig. 3b). In particular, the new method produced similar curves between wild and mutant templates (Ct: 17.11 vs 17.98), while the conventional method produced an obviously later curve (Ct = 26.35) with lower fluorescent signal for mutant template than for wild template (Ct = 18.77) (Fig. 3b). These results suggest that the amplification mediated by the new method was less affected by variants in target genes, implying a promising application in detection of highly variable pathogens.

On the other hand, since larger amplicon will reduce the sensitivity of the conventional qPCR, the amplicon length is generally recommended to be about 100 bp (50–150 bps)^2^. To evaluate the flexibility of the new qPCR method to amplicon lengths, we designed a series of reverse primers to generate various lengths (from 55 to 250 bps) of amplicons with the HFman probe (Supplementary Table S2), and compared the amplification efficiencies. The results showed the amplification curves of different lengths of amplicons cluster together with a small difference in Ct values (16.32 to 19.04), indicating that the amplicon length has less influence on the sensitivity of the new qPCR method (Supplementary Fig. S4).

### Application of the new RT-qPCR in quantification of gene expression

**Figure 4:**
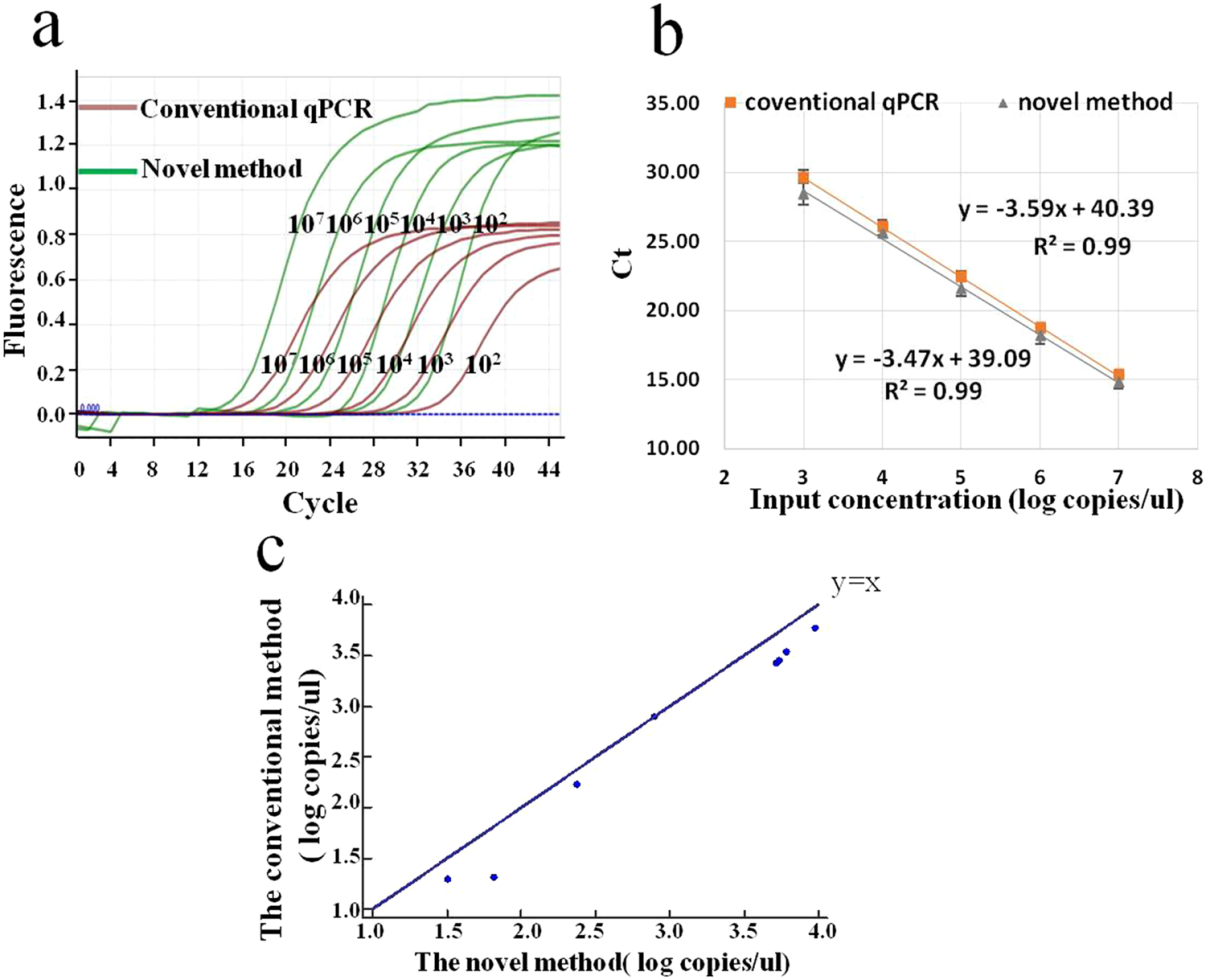
Comparison of the novel and conventional RT-qPCR methods in quantification of *β-actin* gene expression. (**a**) Amplification curves using ten-fold serial dilutions of RNA standard from 10^7^–10^2^ copies/µl. (**b**) Standard curves. Error bars are based on triplicate wells. A comparison between the novel and the conventional qRT-PCR was performed using the Student's t-test and statistical significance is indicated as *P < 0.05; or **P < 0.01. (**c**) Comparison of *β-actin* mRNA among eight healthy blood samples by two methods. The novel RT-qPCR used 400 nM ACTB-R1 and 100 nM ACTB-probe-1, and the conventional RT-qPCR method used 400 nM ACTB-F2 and ACTB-R1 and 100 nM ACTB-probe-1. The blue lines represent the diagonal lines.

To test the application of the new RT-qPCR in quantification of gene expression, we established RT-qPCR assays based on the new method (HFman probe) and the conventional method (TaqMan probe) to quantify the expression of *β-actin*. The RNA standards were prepared from 1×10^3^ to 1×10^7^ copies/µl. Compared with the conventional method (Ct: 15.31 to 29.63), the new method produced amplification curves with relatively lower Ct values (14.76 to 28.44) and obviously stronger fluorescent signals, suggesting a higher sensitivity of the new method (Fig. 4a). The standard curves generated by both methods showed wide dynamic range scope from 1×10^3^ to 1×10^7^ copies/μl and consistent linear correlation coefficient (R^2^ = 0.99) (Fig. 4b). Then, we used both methods to quantify the expression of *β-actin* among 8 blood samples from healthy individuals. Although the expression levels obtained by both methods are well linear correlation (R^2^ = 0.99), the results of the new method were slightly higher than those by the conventional RT-qPCR method (Fig. 4c).

### Application of the new RT-qPCR in quantification of HIV viral load

**Figure 5:**
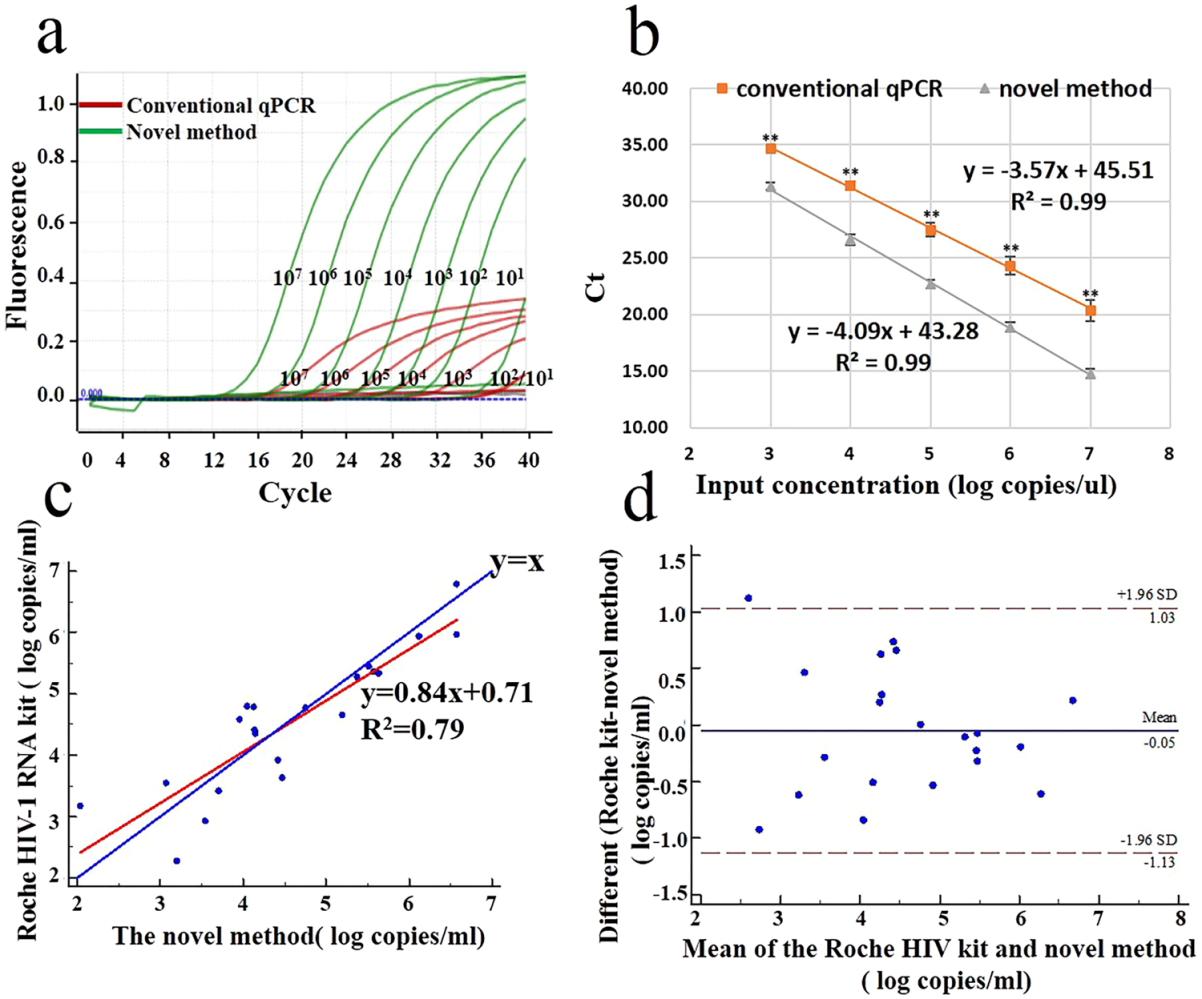
Comparison of the novel and conventional RT-qPCR methods in quantification of HIV-1 viral load. (**a**) Amplification curves using ten-fold serial dilutions of HIV-1 RNA standard from 10^7^–10^0^ copies/µl. (**b**) The standard curves. Error bars are based on triplicate wells. A comparison between the novel and the conventional RT-qPCR was performed using the Student’s t-test and statistical significance is indicated as *P < 0.05; or **P < 0.01. (**c**) Comparison HIV-1 viral loads obtained by the novel method and the Roche HIV-1 RNA kit. A total 21 clinical samples were used. The red and blue lines represent the regression and diagonal lines, respectively. (**d**) Bland Altman plot to assess the agreement between the novel assay and the Roche HIV-1 kit in 21 clinical samples.

HIV-1 is one of the most genetically variable human viruses. To test the application of the new RT-qPCR in virus detection, we established two assays to quantify HIV-1 viral load using the new and conventional methods. Well standard curves with wide linear ranges were obtained by both methods. The assay based on the new method showed about one order of magnitude higher sensitivity than that based on the conventional method (Fig. 5a,b). The accuracy and specificity of this method were demonstrated by sequencing the amplicon and alignment with HIV-1 reference sequence (Supplementary Fig. S5). Using both assays, we detected seven HIV-1 positive samples. All 7 samples (100%) were detected as positive by the new method, while only 4 (57%) were detected as positive by the conventional method (data not shown), possibly due to low viral load. This result suggested that the HFman probe-based RT-qPCR has higher sensitivity than the TaqMan probe-based conventional RT-qPCR.

To further evaluate the performance of the new method in quantification of HIV-1 viral load, we compared it with the COBAS® AmpliPrep /COBAS® TaqMan® HIV-1 Test, v2.0 using 21 HIV-positive plasma samples. The 21 HIV-1 positive samples covered B, CRF01_AE, CRF07_BC, and CRF08_BC subtypes. All 21 HIV samples were detected as HIV RNA positive by our new method and the Roche HIV-1 RNA detection (Fig. 5c). HIV-1 viral load of these samples ranged from 189 to 6010000 copies/ml by the Roche kit, and from 110 to 3781285 copies/ml by our new method. Among 21 samples, 12 samples (57.1%) had higher viral load by our new method than the Roche kit, and eight samples (38.1%) had opposite results (Fig. 5c). The observed relationship between both assays was linear with a good correlation coefficient (R^2^ = 0.79) (Fig. 5c). Bland–Altman analysis estimated the 95% limits of agreement between the two methods within the range of −1.13 to +1.03 log (copies/ml) (Fig. 5d).

### Application of the new RT-qPCR in the detection of Aldehyde dehydrogenase 2 SNP

We previously developed a modified proof-reading (PR-) PCR to detect mutations by combining Taq DNA polymerase with a trace amount of HF DNA polymerases^35^. As shown above and other previous studies^34, 35^, the PR-PCR mediated only by HF DNA polymerase is unable to efficiently distinguish the wild and mutant template. In order to improve the application of the new RT-qPCR in single nucleotide polymorphism (SNP) detection, we reconstructed the new RT-qPCR reaction by adding 2.5 U Taq DNA polymerase (Tiangen, Beijing, China) and 0.1 U Q5 HF DNA polymerase. The concentrations of HFman probe and reverse primer were 100 and 400 nM, respectively.

**Figure 6:**
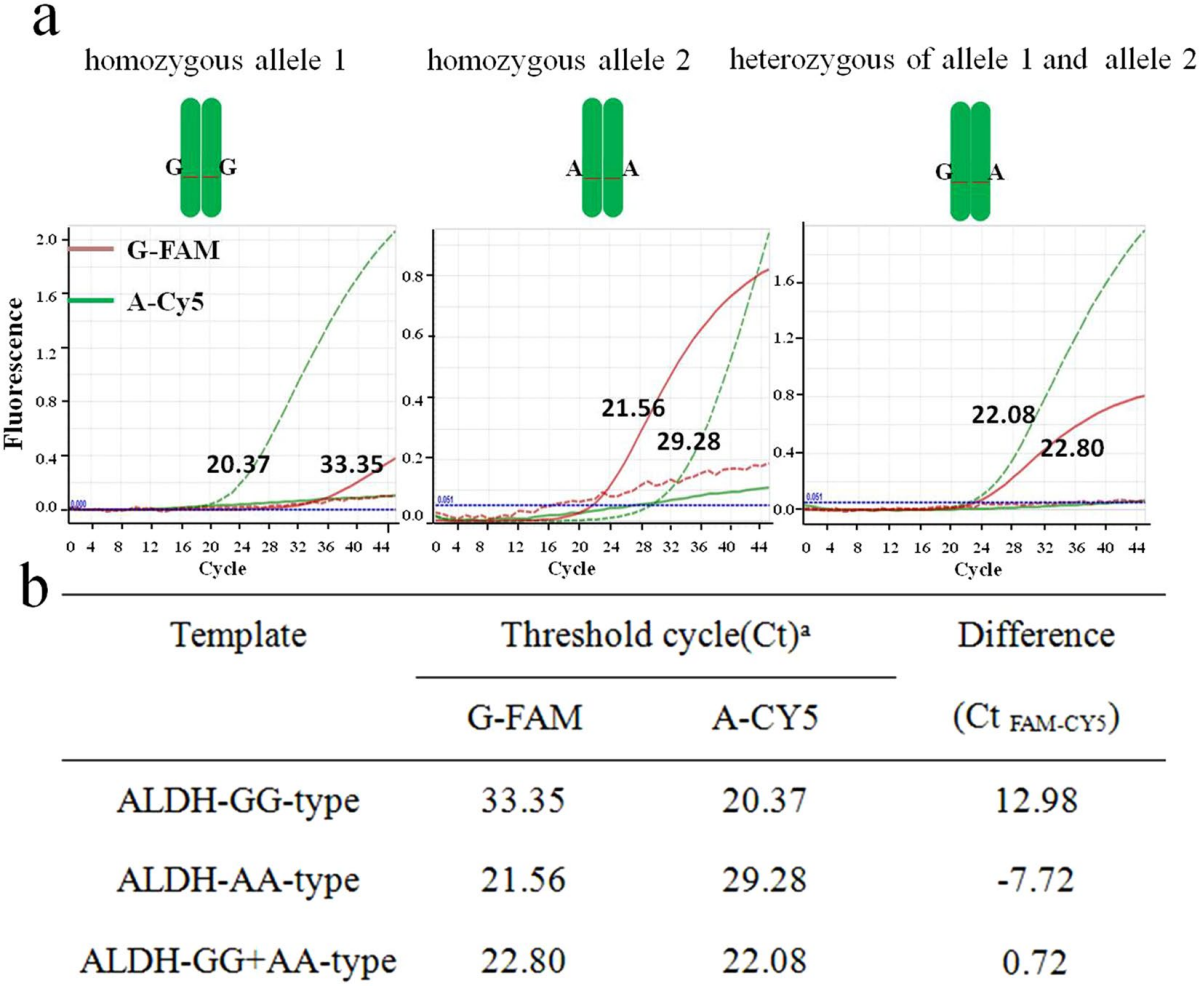
Detection of ALDH2 SNP rs671 using the novel qPCR with a mix of Taq and HF DNA polymerases. (**a**) Amplification curves for three genotypes GG, AA and GA. (**b**) The Ct values of three genotypes. For each samples, two reaction with G-FAM and A-CY5 probes were performed. Probes G-FAM and A-CY5 fully match with homozygous alleles GG and AA, respectively.

Aldehyde dehydrogenase 2 (ALDH2) oxidizes aldehyde to acetic acid mainly in the mitochondria and is a crucial enzyme in alcohol metabolism. There is a SNP (SNP rs671) with G→A substitution in exon 12 of ALDH2 gene that causes a mutation of Glu487Lys and inactivates the enzyme activity^36^. We designed two allele-specific HFman probes (ALDH-probe-G and ALDH-probe-A) and a reverse primer for the detection of SNP rs671 of ALDH2. ALDH-probe-G and ALDH-probe-A were labelled by FAM (red) and CY5 (green), and completely match to homozygous wild (GG) and mutant (AA) alleles, respectively. According to the principle, the RT-qPCR with ALDH-probe-G can amplify the mutant type (AA), but not or inefficiently amplify the wild type of ALDH2 (GG). In contrast, the reaction with ALDH-probe-A can amplify the wild type (GG), but not or inefficiently amplify the mutant type of ALDH2 (AA). Both reactions can amplify the heterozygous type (GA). Comparison of the amplification curves showed that the curve with ALDH-probe-A was obviously earlier than that with ALDH-probe-G for homozygous allele GG, later for homozygous allele AA and similar to each other for heterozygous GA (Fig. 6). Therefore, the difference in Ct values was recommended to identify gene type. We calculated the difference in Ct value by the formula Ct-FAM minus Ct-CY5. The alleles GG, AA and GA were clearly distinguished by the Ct differences of 12.98, −7.72 and 0.72, respectively (Fig. 6). These results suggest that the new RT-qPCR can also be developed to efficiently detect SNP.

## Discussion

Epidemic of various infectious diseases seriously threaten global public health^14, 37^. Rapid diagnosis of involved pathogens plays a crucial role in the prevention and control of infectious diseases^3, 38^. Various new methods were developed for the detection and diagnostics of infectious diseases in the last two decades^39^. However, because of low sensitivity, low specificity, time-consuming and labor-consuming, and high costs, the practical application and commercial development of most methods are limited. To date, TaqMan probe-based qPCR is still the most robust method used in the detection and diagnostics of infectious diseases^14^.

Conventional TaqMan probe-based qPCR is mediated by Taq DNA polymerase and requires a pair of primers and a probe^18^. To archive highly sensitivity and broad spectrum for highly heterogenetic viruses, the primers and probe should target the most conserved genomic regions of the involved viruses^29^. Furthermore, the TaqMan probe-based qPCR often limits the amplicon length between 50–150 bps to achieve high amplification efficiency^2^. For highly heterogenetic viruses, however, it is very difficult to find three completely conserved regions in a relative short genomic region that satisfy the criteria of primer and probe design. Although degenerate primers and MGB-modified probe were used to reduce the influence of mismatches of primer or probe with target on amplification efficiency, to develop highly accurate, sensitive and board spectrum detection method for highly variable viruses is still a big challenge^29^.

In this study, we developed a novel, simple real-time qPCR mediated by high-fidelity DNA polymerase. Different from the conventional qPCR, the new qPCR only needs one primer and an HFman probe (Table 1). The generation of fluorescent signal was mediated by HF DNA polymerase that separates the fluorophore and quencher by the 3′-5′ hydrolysis of HFman probe. In principle, the HF DNA polymerase only removes the mismatched nucleotide from the primer or probe by its 3′-5′ exonuclease activity (Table 1). However, majority of the HF DNA polymerases show non-specific 3′-5′ exonuclease activity and also remove the completely matched 3′-base of HFman probe possibly due to the modification of fluorophore or quencher activate the 3′-5′ exonuclease activity of HF DNA polymerase (Fig. 1)^34^. Therefore, regardless of match or mismatch between HFman/primer and target template, the new qPCR exhibits similar amplification efficiencies (Fig. 3a), indicating that the new method adapts well to the amplification of various mutation templates. These imply that the new HFman probe-based method is more efficient for the detection of highly variable viruses than the conventional TaqMan probe-based qPCR. Additionally, the new method has similar amplification efficiencies for different sizes (from 55 to 250 bps) of amplicons (Supplementary Fig. S4), and does not require an HFman with 10 °C higher Tm value than that of primers (Table 1). These make the primer and probe design easier and more flexible because the new method requires one HFman probe and one primer, whereas the conventional qPCR method requires one TaqMan probe and a pair of primers (Table 1). One major limitation in the primer and probe selection for the new method is that it does not allow the formation of primer dimers which may result in non-specific result.

**Table 1:** The comparison of the HFman probe-based qPCR with Taqman probe-based qPCR.

Features	Taqman probe-based qPCR	HFman probe-based qPCR
Primer and probe	One pair of primers and one probe (TaqMan)	One primer and one probe (HFman)
Enzyme	Taq DNA polymerase	High fidelity DNA polymerase
Tolerance to mismatch between primer/probe and template (variable templates)	No, not allow mismatch	Yes, the mismatch initiates better elongation.
Optimal amplicon size (bps)	50–150 bps	55–250 bps
Optimal length of probe	25–32 nt	20–30 nt
Tm value of Taqman probe	68–70 °C (at least 10 °C higher than that of the primers)	> 55 °C (similar to that of the primer)
Primer dimers	Allowance	No allowance

To evaluate the performance of the new HF DNA polymerase mediated qPCR, we quantified *β-actin* expression and HIV-1 viral load using the new method and compared it with the conventional method. The results showed that the new method had slightly higher sensitivity than the conventional method (Figs 4 and 5). Of particular importance is that the new method can amplify various variants with a similar amplification efficiencies to wild type template, better than the conventional qPCR method that had an obviously lower amplification efficiency for variants than for the wild-type template (Fig. 3b).

HIV is the most genetically variable virus ever studied^31^. We developed a HIV-1 assay with a limit of detection (LOD) of 23 copies/ml using the new method (Supplementary Table S3). To evaluate the compatibility of our HIV-1 assay, we performed a comparison with COBAS® AmpliPrep /COBAS® TaqMan® HIV-1 Test, v2.0, the most widely used kit worldwide with highest detection sensitivity for HIV-1 quantification, using 21 HIV-1 positive clinical samples covering HIV-1 subtypes B, CRF01_AE, CRF07_BC, and CRF08_BC. All these samples could be detected by the Roche kit (VL: from 189 to 6010000 copies/ml) and our new method (VL: from 110 to 3781285 copies/ml) (Fig. 5c), and a good correlation coefficient (R^2^ = 0.79) was observed between both methods (Fig. 5c). In particular, our method detected 12 sample with higher viral loads than the Roche kit, possibly due to the well adaptation of our method to various variants. These results suggest that the HF DNA polymerase mediated RT-qPCR is comparable to the Roche kit and/or other commercial HIV-1 kits for HIV-1 quantification (Supplementary Table S3). In addition, we established assays for H7N9 (N9 gene), and human coronavirus NL63, and obtained well amplification and standard curves (Supplementary Fig. S6), indicating that the new method is suitable for the detection of other viruses. HF DNA polymerase can mediate the proof-reading (PR-) PCR for SNP and mutation detection^34^. We previously developed a modified PR-PCR using a ddNTP-blocked primer and a mixture of Taq DNA polymerases and HF DNA polymerase to high-efficient detection of various mutations^35^. Based on the similarity in the principle, we modified the new method by using an enzyme mixture of 2.5 U Taq DNA polymerase and 0.1 U Q5 DNA polymerase and extended its application to SNP detection. A good performance was observed when SNP rs671 of ALDH2 was detected (Fig. 6).

In summary, we developed a high-fidelity DNA polymerase-mediated qPCR method that utilizes one HFman probe and one primer. The new qPCR exhibits higher detection sensitivity and better adaptability to variable templates than the conventional TaqMan probe-based qPCR (Table 1). In addition, the probe and primer design for the new method is easier and more flexible than the conventional method. Therefore, the high-fidelity DNA polymerase-mediated qPCR method is a simple, sensitive and promising approach for molecular diagnosis, especially for diagnostics of infectious diseases.

## Materials and Methods

### Oligonucleotides design

Primers were designed using the Primer Premier 5.0 program. Like a TaqMan probe, the HFman probe was designed in general with a fluorophore and a quencher at 5′ and 3′ end, respectively; however, a better form of the HFman probe is to have a 3′ fluorophore and a 5′ quencher. All primers were synthesized commercially by Thermofisher scientific (Shanghai, China). The detailed information of the primers, TaqMan and HFman probes are shown in Supplementary Tables S1 and S2.

### Preparation of RNA standard and RNA templates with various mutants

Various target gene fragments (human genes or virus genomic sequences) containing a T7 promoter were obtained by gene synthesis or RT-PCR amplification, and then cloned into pMD18-T plasmids (TaKaRa, Dalian China). A series of mutants were prepared using Fast Mutagenesis System (Transgene Biotech, Beijing, China). To obtain RNA template, *in vitro* transcription were performed using the recombinant plasmids or amplification products of the plasmids. RNA was quantified, serially diluted and used as RNA standard or templates.

### The novel and conventional real-time RT-PCR reactions

The novel (using HFman probe) and conventional (using TaqMan probe) real-time RT-PCR reactions were carried out in a Light Cycler 96 Real-Time PCR System (Roche Diagnostics, Germany) using the Q5® Hot Start High-Fidelity 2× Master Mix (New England Biolabs, American), and Quant One Step qRT-PCR Kit (Tiangen Biotechnology Co., Ltd, Beijing, China), respectively. The assays were performed in 25 µl of reaction with 3 µl of RNA or DNA. For the novel assay, the reaction contains 12.5 µl of Q5® Hot Start High-Fidelity 2× Master, 0.35 μl AMV RTase (10 U/μl) (Sangon Biotech Co., Ltd,Shanghai, China), 100 nM HFman probe, and 400 nM normal primer (reverse primer). The conventional assay contains 12.5 μl 2× Quant One Step Probe qRT-PCR Master Mix, 1 μl HotMaster Taq polymerase (2.5 U/μl), 0.35 μl Quant RTase, 400 nM each forward and reverse primer, 100 nM TaqMan probe. The cycling condition was performed as following: RT at 50 °C for 30 min, enzyme activation at 92 °C for 3 min, followed by 45 cycles of denaturation at 92 °C for 10s, annealling and extension at 55 °C for 30s. Fluorescence signal was collected at the annealling and extension step of each cycle. The threshold value was set to 0.05 automatically.

### Application of the novel method in quantification of gene expression

To prove the availability of the novel method in gene expression quantification, we used the method to detect the expression of *β-actin* gene among white blood cells from eight healthy individuals. Comparison was performed with the conventional RT-qPCR with TaqMan probe. The whole blood samples of healthy individuals were obtained from Taizhou Fourth People’s Hospital, Jiangsu, China. The mRNA was extracted from 200 μl whole blood using the PureLink RNA Mini Kit (Thermo Fisher Scientific, Massachusetts, USA) according to the manufacturer’s instruction and eluted in 60 µl nuclease-free water. The RT-qPCR reactions were performed as described above.

### Application of the novel method in HIV-1 RNA quantification and comparison with conventional RT-qPCR and a commercial HIV-1 kit

To prove the availability of the novel method in virus detection, we designed HFman and TaqMan probes and develop corresponding RT-qPCR methods for HIV-1 RNA quantification. The HFman, TaqMan probes and corresponding primers are located in HIV-1 integrase gene (Supplementary Table S1). In order to evaluate the performance of the novel method in HIV-1 quantification, we performed a comparison with a commercial kit COBAS® AmpliPrep /COBAS® TaqMan® HIV-1 Test, v2.0 (Roche Diagnostics, Germany) that is most widely used in the world using 21 HIV positive plasma samples. The samples were obtained from Shanghai Municipal Center for Disease Control & Prevention, China. HIV-1 RNA was extracted from 140 µl plasma and eluted into 60 µl AVE buffer using QIAamp Viral RNA Mini Kit (Qiagen, Germany). The viral load by the commercial kit was calculated according to the manufacturer’s instruction. The viral load by the novel method was calculated using the formula: Copy number per reaction * (60/3) * (1000/140). A Bland–Altman analysis was used to assess the level of agreement between the commercial kit and novel method. A range of agreement was defined as the mean of difference (bias) ±1.96 SD.

### Ethics statement

The use of clinical samples and the experiments involved in these sample were approved by the Institutional Review Board at the Human Medical Research Ethics Committee of the Shanghai Municipal Center for Disease Control and Prevention. Quantification of HIV-1 viral load was carried out in accordance with the guideline and regulation of the National HIV Reference Laboratory, China Center for Disease Control and Prevention. Informed consent was obtained from all subjects.

## Electronic supplementary material

supplementary information
