# Author Correction: A novel quantitative PCR mediated by high-fidelity DNA polymerase

**DOI:** 10.1038/s41598-018-24093-9

**Published:** 2018-04-12

**Authors:** Mengling Zhang, Kyle Liu, Yihong Hu, Yi Lin, Yang Li, Ping Zhong, Xia Jin, Xiaoli Zhu, Chiyu Zhang

**Affiliations:** 1 School of Life Sciences, Shanghai University, Shanghai, China; 2 Pathogen Diagnostic Center, CAS Key Laboratory of Molecular Virology & Immunology, Institut Pasteur of Shanghai, Chinese Academy of Science, Shanghai, China; 3 Shanghai Municipal Center for Disease Control & Prevention, Shanghai, China; 4 Viral Disease and Vaccine Translational Research Unit, CAS Key Laboratory of Molecular Virology & Immunology, Institut Pasteur of Shanghai, Chinese Academy of Sciences, Shanghai, China

Correction to: *Scientific Reports* 10.1038/s41598-017-10782-4, published online 04 September 2017

This Article contains typographical errors in the Acknowledgements section.

“National Natural Science Foundation of China (U13022081)”

should read:

“National Natural Science Foundation of China (U1302224)”.

