# Supplementary material for: A novel quantitative PCR mediated by high-fidelity DNA polymerase
Source: Sci Rep. 2017 Sep 4;7:10365. doi: 10.1038/s41598-017-10782-4 (PMC5583327; doi:10.1038/s41598-017-10782-4)
Supplement: Supplementary file 1 — supplementary information [file 41598_2017_10782_MOESM1_ESM.pdf]

A novel quantitative PCR mediated by high-fidelity DNA polymerase

Mengling Zhang[1,2], Kyle Liu[1], Yihong Hu[2], Yi Lin[3], Yang Li[2], Ping Zhong[3], Xia Jin[4], Xiaoli Zhu[1], Chiyu Zhang[2,*]

1 School of Life Sciences, Shanghai University, Shanghai, China

2 Pathogen Diagnostic Center, CAS Key Laboratory of Molecular Virology & Immunology, Institut Pasteur of Shanghai, Chinese Academy of Science, Shanghai, China

3 Shanghai Municipal Center for Disease Control & Prevention, Shanghai, China

4 Viral Disease and Vaccine Translational Research Unit, CAS Key Laboratory of Molecular Virology & Immunology, Institut Pasteur of Shanghai, Chinese Academy of Sciences, Shanghai, China.

Table S1. The sequence information of oligonucleotides used

Targets	Primer/probe/template[a]	Sequence (5'-3')
H7N9	N9-probe-1	FAM-ATCATCACCGCCCACAGTGTACAA-BHQ1
	N9-probe-2	BHQ1-ATCATCACCGCCCACAGTGTACAA-FAM
	N9-R1	ACATCCTGGATTTGCCATCA
β-actin	ACTB-probe-1/A	HEX-TGGACTTCGAGCAAGAGATGGCCA-BHQ1
	ACTB-probe-2	CY5- TGGACTTCGAGCAAGAGATGGCCA-BHQ3
	ACTB-probe-3	TexRed-TGGACTTCGAGCAAGAGATGGCCA-ECLIPSE
	ACTB-probe-4	FAM-TGGACTTCGAGCAAGAGATGGCCA-TAMRA
	ACTB-probe-5	HEX-CATCACCATTGGCAATGAGCGGTT-BHQ1
	ACTB-probe-G	HEX-TGGACTTCGAGCAAGAGATGGCCG-BHQ1
	ACTB-probe-T	HEX-TGGACTTCGAGCAAGAGATGGCCT-BHQ1
	ACTB-probe-C	HEX-TGGACTTCGAGCAAGAGATGGCCC-BHQ1
	ACTB-F1	TGGACTTCGAGCAAGAGATGGCCA
	ACTB-F2	GGAAATCGTGCGTGACATTAAG
	ACTB-R1	ATTGCCAATGGTGATGACCTG
	ACTB-R2	CAGGAAGGAAGGCTGGAAGA
The templates of *β-actin*	ACTB-WT	TGGACTTCGAGCAAGAGATGGCCA
	ACTB-Mu1	TGGACTTCGAGCAAGAGATGGCC**G**
	ACTB-Mu2	TGGACTTCGAGCAAGAGATGGC**G**A
	ACTB-Mu3	TGGACTTCGAGCAAGAGATGGC**T**A
	ACTB-Mu4	**T**AGGTCATCACCATTGGCAAT
	ACTB-Mu5	**G**AGGTCATCACCATTGGCAAT
	ACTB-Mu6	TGGACTTCGAGCAAGAGATGGCC**T**
	ACTB-Mu7	TGGACTTCGAGCAAGAGATGGCC**C**
HIV-1	HIV-probe-1	BHQ1-GGGGTACAGTGCAGGGGAAAGAA-FAM
	HIV-F1	GACAGCAGTACAAATGGCAG
	HIV-R1	TAAACCCGAAAATTTTGAATT

ALDH	ALDH-probe-G	FAM-GAGTACGGGCTGCAGGCATACACTG-BHQ1
	ALDH-probe-A	CY5-GAGTACGGGCTGCAGGCATACACTA-BHQ1
	ALDH-R1	CGAGCCACCAGCAGACCCTC
NL63	NL63-probe	FAM-TCAGTGCTCGCTTCAACAACACAACCA-BHQ1
	NL63-R	CAGTTATGATTTCACAATGGCCT

[a] For the names of oligonucleotides: F, forward primer; R, reverse primer; WT, wild-type; Mu, mutant.

[b] Bold characters indicate the point mutation.

Table S2. Sequences information of ACTB primers and the corresponding amplicon sizes

Name of oligonucleotides	Sequence (5'-3')	Sizes of amplicons
ACTB-probe-1	HEX-TGGACTTCGAGCAAGAGATGGCCT-BHQ1	
ACTB-R1	ATTGCCAATGGTGATGACCTG	95 bp
ACTB-R2	CAGGAAGGAAGGCTGGAAGA	140 bp
ACTB-R3	CTCTTCTCCAGGGAGGAGCT	55 bp
ACTB-R4	GTTTCGTGGATGCCACAGG	169 bp
ACTB-R5	GAGTTGAAGGTAGTTTCGTGGA	181 bp
ACTB-R6	CACGTCACACTTCATGATGG	200 bp
ACTB-R7	GTGGTGCCGCCAGACAGCAC	250 bp

[a] For the names of oligonucleotides: R, reverse primer;

Table S3. Comparison of sensitivity between the novel method and other commercial HIV-1 kits

Method	Target gene	Amplification	Sample volume	LOD	Reference
HFman PCR	*Integrase*	HFman probe based-qPCR	1 ml	23 copies/ml *	This study
CAP/CA v1.5	*gag*	TaqMan probe based-qPCR	0.5 ml	50 copies/ml	1
CAP/CTM v1.0	*gag*	TaqMan probe based-qPCR	1.0 ml	48 copies/ml	2
CAP/CTM v2.0	*gag* and LTR	TaqMan probe based-qPCR	1.0 ml	20 copies/ml	3
Aptima HIV	*pol* and LTR	transcription mediated amplification(TMA)	0.5 ml	30 copies/mL	4
Abbott HIV-1	*Integrase*	Double-stranded probe based-qPCR	1.0 ml	40 copies/ml	5

LOD, Limit of detection; CAP/CA v1.5, COBAS® AMPLICOR HIV-1 MONITOR Test, v1.5; CAP/CTM v1.0, COBAS® AmpliPrep/COBAS® TaqMan® HIV-1 Test, v1.0; CAP/CTM v2.0, COBAS® AmpliPrep/COBAS® TaqMan® HIV-1 Test, v2.0; Aptima HIV, Hologic Aptima HIV-1 Quant Dx assay; Abbott real-time HIV-1, Abbott m2000 RealTime HIV-1 assay.

* The LOD of the assay is 23 copies per 25 μl reaction. If 0.5-1 ml plasma is used for producing 50 μl RNA solution, and all RNA solution is used in a 100 μl reaction, the sensitivity of our HIV-1 assay can reach 46-23 copies/ml.

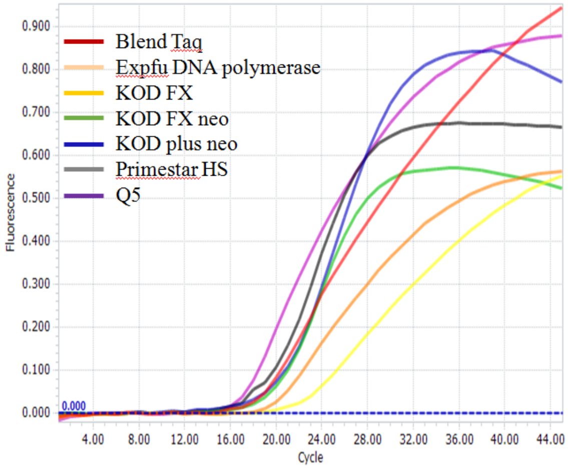

Fig. S1 Performance of the novel qPCR methods using various high-fidelity DNA polymerases. Seven high-fidelity DNA polymerases including Blend Taq, Expfu DNA polymerase, KOD FX, KOD FX neo, KOD plus neo, Primestar HS, and Q5 were used detect *β-actin* RNA. The corresponding buffer was used for each enzyme with additional add of 1μl 75mM $MgCl_2$. The real-time fluorescence curve of *β-actin* RNA produced by ACTB-probe-1 and ACTB-R1.

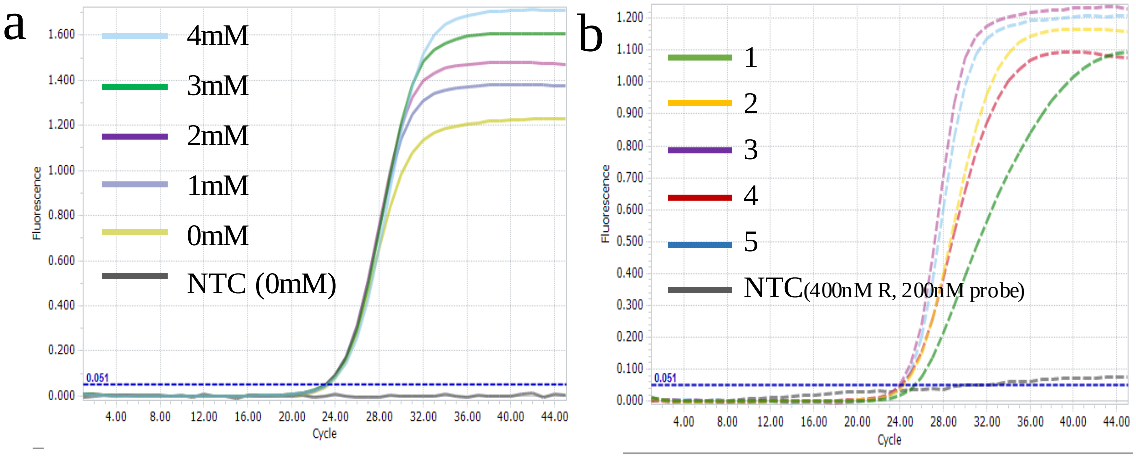

Primer and probe	Concentration(μM)				
	1	2	3	4	5
R(μM)	0.4	0.4	0.4	0.2	0.2
Probe(μM)	0.4	0.2	0.1	0.2	0.1

Fig. S2 Optimization of reaction condition of the novel method. (a) The influence of $MgCl_2$ concentration on amplification. (b) The influence of primer and probe concentration of primer and probe on amplification. NTC, non-template control. The real-time fluorescence curve produced by *β -actin* RNA with ACTB-probe-1 and ACTB-R1.

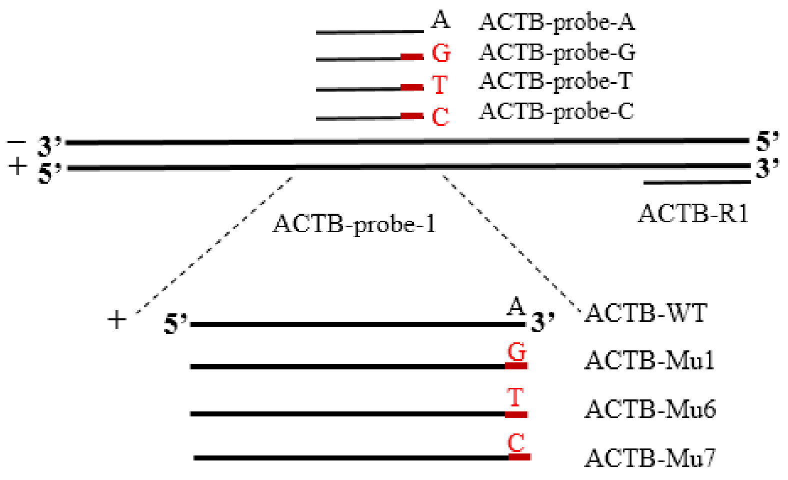

Template	Threshold cycle (Ct)[a]			
(RNA 10^4)	ACTB-probe-A	ACTB-probe-G	ACTB-probe-T	ACTB-probe-C
ACTB-WT	24.32	23.79	24.06	23.56
ACTB-Mu1	25.02	25.38	25.26	25.35
ACTB-Mu6	24.89	24.57	24.39	25.15
ACTB-Mu7	26.03	25.62	25.66	27.58

Fig. S3 The influence of various mismatches between probe/primer and template on the novel qPCR. The probes were labelled by HEX and BHQ1 at 5' and 3' ends, respectively. 10^4 RNA of *β-actin* RNA standard was used in the experiments. For the sequence information of probes and primers, please see Supplementary Table S1.

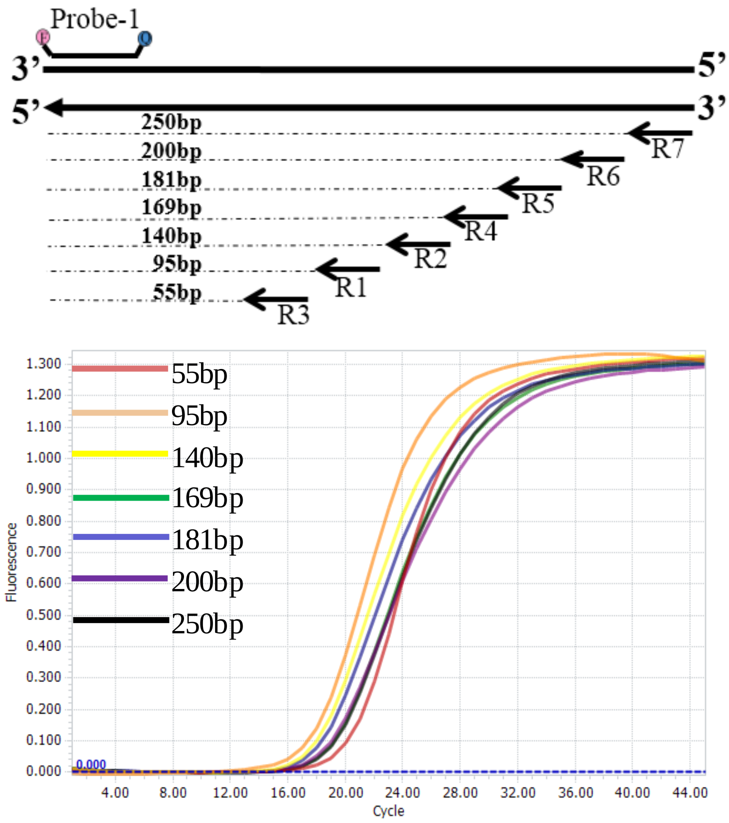

Fig. S4 Influence of different sizes of amplicons on the amplification of the novel method. It was conducted by ACTB-probe-1 and ACTB-R1 to detect *β-actin* RNA. For the sequence information of probes and primers, please see Supplementary Table S1.

HIV-1

Target sequence: GACAGCAGTACAAATGGCAGTATTCATCCACAATTTTAAAAGAAAAGGGGGGATTGGGGGGTACAGTGCAGGGGAAAGAATAGTAGACATAATAGCAACAGACATACAAACTAAAGAAT

Sequence1(TaqMan): GACAGCAGTACAAATGGCAGTATTCATCCACAATTTTAAAAGAAAAGGGGGGATTGGGGGGTACAGTGCAGGGGAAAGAATAGTAGACATAATAGCAACAGACATACAAACTAAAGAAT

Sequence2(HFman): GGGGTACAGTGCAGGGGAAAGAATAGTAGACATAATAGCAACAGACATACAAACTAAAGAAT

β-actin

Target sequence: GGAAATCGTGCGTGACATTAAGGAGAAGCTGTGCTACGTCGCCCTGGACTTCGAGCAAGAGATGGCCACGGCTGCTTCCAGCTCCTCCCTG

Sequence1(TaqMan): GGAAATCGTGCGTGACATTAAGGAGAAGCTGTGCTACGTCGCCCTGGACTTCGAGCAAGAGATGGCCACGGCTGCTTCCAGCTCCTCCCTG

Sequence2(HFman): TGGACTTCGAGCAAGGTTTGGCCACGGCTGCTTCCAGCTCCTCCCTG

Fig. S5 The sequence alignments of amplicons of the new (HFman) and conventional (TaqMan) qPCR. The new and conventional methods share reverse primer. The amplicon of the new method is smaller than that of the conventional method.

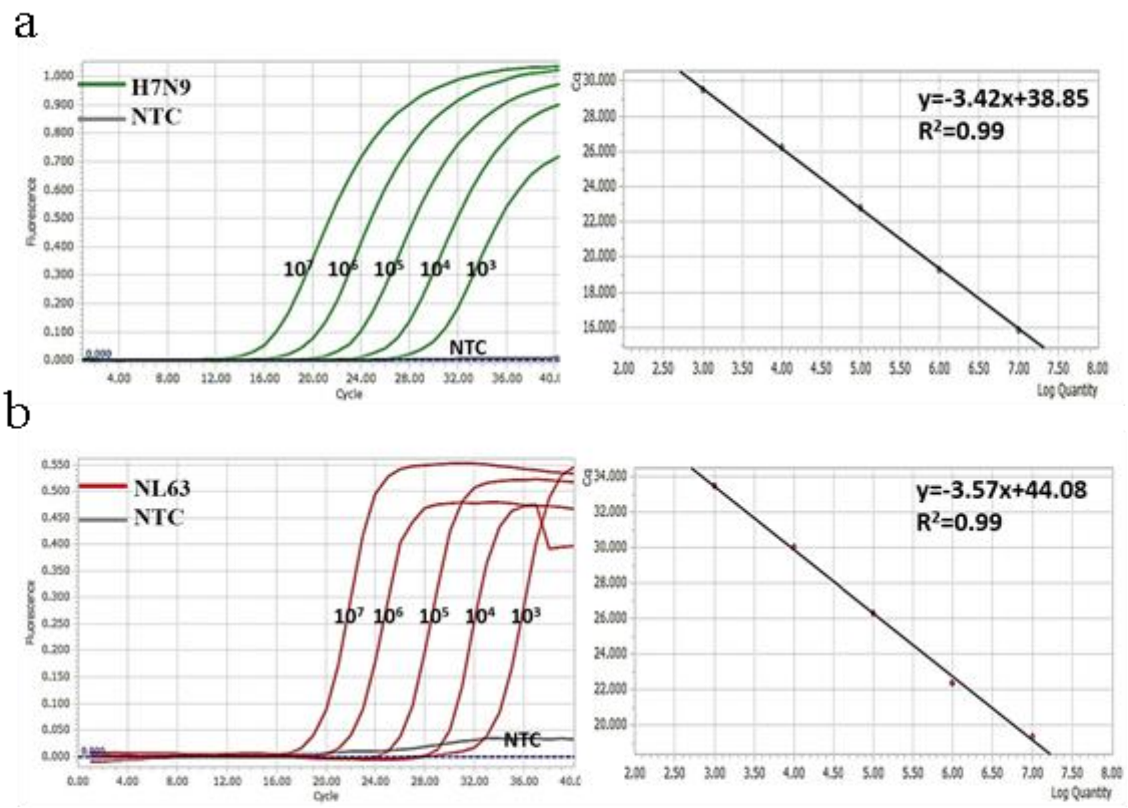

Fig. S6 Amplification and standard curves of H7N9 (a) and human coronavirus NL63 (b) using the new method. Amplification curves and standard curves were obtained using ten-fold serial dilutions of RNA standard from 10^7-10^3copies/µl. The concentrations of HFman probe and reverse primer are 100 nM and 400 nM, respectively.

References

1. Lee, B. G., Fiebelkorn, K. R., Caliendo, A. M., & Nolte, F. S.. Development and verification of an automated sample processing protocol for quantitation of human immunodeficiency virus type 1 RNA in plasma. *Journal of clinical microbiology*. **41,** 2062-2067 (2003).
2. Schumacher W. et al. Fully automated quantification of human immunodeficiency virus (HIV) type 1 RNA in human plasma by the COBAS® AmpliPrep/COBAS® TaqMan® system. *Journal of Clinical Virology*. **38,** 304-312 (2007).
3. Sizmann, D. et al. Improved hiv-1 rna quantitation by cobas ampliprep/cobas taqman hiv-1 test, v2.0 using a novel dual-target approach. *Journal of Clinical Virology*. **49,** 41-46 (2010).
4. Manak, M. et al. Evaluation of the Hologic (Gen-Probe) Aptima HIV-1 RT Quantitative Assay on HIV-1 Subtypes.
5. Michael T. Pyne , Eric Q. Konnick , Amit Phansalkar & David R. Hillyard. Evaluation of the Abbott RealTime HIV-1 Assay.
